# Characteristics of Tau and Its Ligands in PET Imaging

**DOI:** 10.3390/biom6010007

**Published:** 2016-01-06

**Authors:** Ryuichi Harada, Nobuyuki Okamura, Shozo Furumoto, Tetsuro Tago, Kazuhiko Yanai, Hiroyuki Arai, Yukitsuka Kudo

**Affiliations:** 1 Division of Neuro-imaging, Institute of Development, Aging and Cancer, Tohoku University, 4-1 Seiryo-machi, Aoba-ku, Sendai 980-8575, Japan; dragon1@med.tohoku.ac.jp (R.H.); kudoyk3y7k3@med.tohoku.ac.jp (Y.K.); 2 Department of Pharmacology, Tohoku University School of Medicine, 2-1 Seiryo-machi, Aoba-ku, Sendai 980-8575, Japan; yanai@med.tohoku.ac.jp; 3 Division of Radiopharmaceutical Chemistry, Cyclotron and Radioisotope Center, Tohoku University, 6-3Aoba, Aramaki, Aoba-ku, Sendai 980-8578, Japan; furumoto@cyric.tohoku.ac.jp (S.F.); tago@cyric.tohoku.ac.jp (T.T.); 4 Department of Geriatrics and Gerontology, Institute of Development, Aging and Cancer, Tohoku University, 4-1 Seiryo-machi, Aoba-ku, Sendai 980-8575, Japan; hiroyuki.arai.b5@tohoku.ac.jp

**Keywords:** Alzheimer’s disease, tau deposits, positron emission tomography, radiotracer

## Abstract

Tau deposition is one of the neuropathological hallmarks in Alzheimer’s disease as well as in other neurodegenerative disorders called tauopathies. Recent efforts to develop selective tau radiopharmaceuticals have allowed the visualization of tau deposits *in vivo*. *In vivo* tau imaging allows the assessment of the regional distribution of tau deposits in a single human subject over time for determining the pathophysiology of tau accumulation in aging and neurodegenerative conditions as well as for application in drug discovery of anti-dementia drugs as surrogate markers. However, tau deposits show complicated characteristics because of different isoform composition, histopathology, and ultrastructure in various neurodegenerative conditions. In addition, since tau radiopharmaceuticals possess different chemotype classes, they may show different binding characteristics with heterogeneous tau deposits. In this review, we describe the characteristics of tau deposits and their ligands that have β-sheet binding properties, and the status of tau imaging in clinical studies.

## 1. Introduction

Tau is a microtubule-associated protein that physically stabilizes microtubule assembly in axons, and pathologically, forms hyperphosphorylated aggregates in the brain in Alzheimer’s disease (AD) and tauopathies. AD is an irreversible and progressive neurodegenerative disease, clinically characterized by cognitive decline, and is the most common cause of dementia in the world. Tauopathies including some variants of frontotemporal lobar degeneration, progressive supranuclear palsy (PSP), corticobasal degeneration (CBD), tangle predominant senile dementia (TPSD), argyrophilic grain disease (AGD), and chronic traumatic encephalopathy (CTE) are also neurodegenerative conditions characterized by the accumulation of tau protein in the brain. CTE is associated with repetitive traumatic brain injury and prevalent among contact sports athletes such as American football players and military personnel victims to blasts in the battlefield [1]. These neuropathological lesions were only established by histopathological analysis such as tau immunohistochemistry at autopsy. Tau deposition occurs in a stereotyped spatiotemporal manner with intraneuronal and neuroanatomical distribution in the brain, widely assessed by Braak staging [2,3]. In addition, the progression of stereotypical regional tau deposition was highly associated with neuronal loss, severity of dementia, and neurodegeneration, unlike amyloid plaque [4,5], which is one of the neuropathological hallmarks in AD [6]. The spread of tau from the medial temporal lobe, such as the entorhinal cortex and hippocampus into the neocortical areas leads to synaptic dysfunction, glial activation, and eventually, neuronal loss, resulting in progressive cognitive impairment [7]. Therefore, understanding the regional distribution of tau deposition seems to be critical for the understanding of clinical presentation. These findings and recent clinical trials failures of anti-amyloid drugs led to an increasing and shifting interest in tau protein as therapeutic targets [8,9]. Ideal treatment for the therapeutic prevention in AD would be to start it before extensive neocortical tau deposition as well as irreversible neuronal loss.

**Figure 1:**
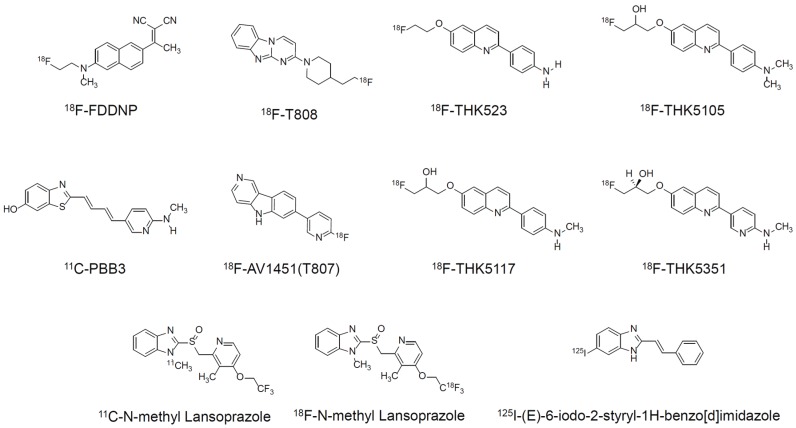
Chemical structures of different chemotype classes of tau ligands.

Positron emission tomography (PET) provides regional and pathophysiological information non-invasively in living human subjects. Use of tau selective radiotracers will enable the noninvasive monitoring of tau pathology in patients with tauopathies, providing better understanding of tau aggregation in the brain. Tau PET imaging would help in the development of anti-tau therapeutics by assisting subject enrollment and evaluation of drug efficacy in clinical trials. Recently, several PET radiotracers with different chemotype classes, such as ^11^C-PBB3, ^18^F-AV1451 (as known as T807), and ^18^F-THK arylquinoline series, for imaging tau deposits were evaluated in humans (Figure 1). The demands of a radiotracer for tau deposits in the brain have already been described in several reviews [10,11,12,13]. The complexity of tau deposits, including their heterogeneous histopathology, isoform composition, and ultrastructural conformations, raises questions about the binding spectrum/sites of tau PET tracers. In addition, since non-AD tau aggregates have different forms that yield different ultrastructures, a single tau PET radiotracer may not detect all of them. In this review, we describe the characteristics of tau deposits, *in vitro* binding characteristics of reported tau PET radiotracers, and status of clinical PET imaging.

## 2. Characteristics of Tau Deposits in Neurodegenerative Conditions

### 2.1. Different Localization and Histopathology of Tau Deposits

**Figure 2:**
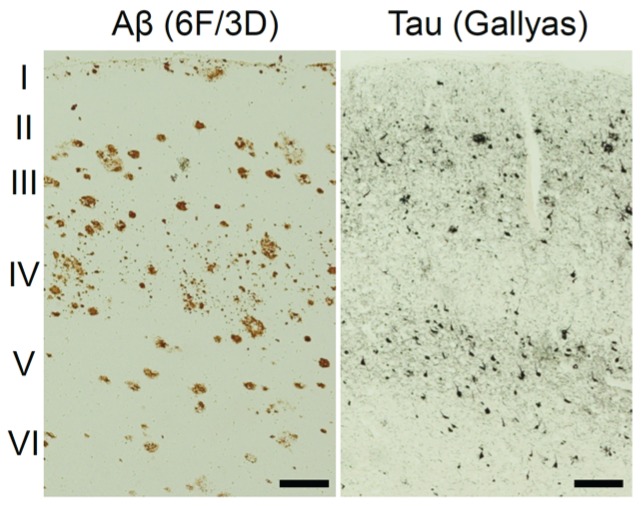
Different distribution of amyloid pathology and neurofibrillary pathology stained with anti-Aβ (6F/3D) antibody and Gallyas silver staining, respectively. Scale bar: 200 μm.

Tau protein plays a role in cytoskeletal support and axonal transport by stabilizing microtubules, which is regulated by the phosphorylation of tau protein [14]. Neuropathological tau deposits in AD are localized in not only axons but also cell bodies and dendritic processes, which are called neurofibrillary tangles and neuropil threads. Tau deposits are also co-localized with amyloid plaques as dystrophic neurites. The neocortex can be divided into six horizontal layers, Layer I–VI. Layer V is the inner pyramidal layer that contains large pyramidal neurons, while layers II and III contain small and medium pyramidal neurons. Postmortem studies showed that tau deposits predominantly appeared in layer V and layers II/III of the neocortex in AD, while amyloid plaques were diffusely distributed in the neocortex (Figure 2) [15,16], indicating that layer-specific tau deposition occurs in AD. Numerous neurofibrillary tangles and neuropil threads were observed in layer V, while dystrophic neurites and neuropil threads were prominent in layers II/III. Pick’s bodies are localized in layer II and layer VI in the frontal and temporal neocortex in Pick’s disease [17]. Although tau deposits in CTE have similar histopathological characteristics as neurofibrillary tangles and neuropil threads, neurofibrillary tangles were observed focally in superficial cortical layers [18,19,20]. In PSP and CBD, tufted astrocytes, astrocytic plaques, coiled bodies, and argyrophilic threads were observed as glial tau deposits [21,22,23]. Tau deposits in CBD and PSP are also observed in the subcortical white matter and brainstem [22]. This evidence shows the distinct localization and histopathological characteristics of tau deposits in neurodegenerative conditions.

### 2.2. Different Isoform Composition of Tau Deposits

Six isoforms of tau protein are produced by alternative splicing of the tau gene and categorized based on the number of microtubule-binding domains into two functionally different groups; three repeat (3R) or four repeat (4R) [24,25]. According to the biochemical analyses of normal adult human brain samples from fresh biopsies, six normal tau isoforms were expressed in the adult brain with approximately equal ratio of 3R and 4R tau isoforms [26]. However, abnormal tau deposits in neurodegenerative conditions contain different isoform compositions; both 3R and 4R tau in AD/TPSD/CTE: predominantly 4R tau in CBD/PSP/AGD and 3R tau in Pick’s disease [20,27,28,29].

### 2.3. Ultrastructure of Filamentous Tau Deposits

Tau aggregates from brain homogenates of patients with AD are highly insoluble in sodium dodecyl sulfate (SDS), urea, reducing agent, and guanidine [30]. X-ray diffraction revealed that tau deposits formed protein filaments having a predominant β-sheet structure that is similar to amyloid plaques [31]. Twisted filaments of hyperphosphorylated tau are called paired helical filaments (PHFs), with a diameter of 8–20 nm and a stereotypical periodicity of 80 nm [32,33]. PHFs account for approximately 95% of the neurofibrillary components in AD, and the rest are composed of straight filaments [33]. TPSD has indistinguishable tau filaments from PHFs in AD [34]. Different ultrastructure of tau deposits have been observed in various neurodegenerative conditions. In both PSP and Pick’s disease, tau deposits are composed of numerous straight filaments, despite different tau isoform compositions [35,36], while tau deposits in CBD are composed of straight filaments and distinct twisted ribbon-like filaments [37]. In frontotemporal dementia with parkinsonism linked to chromosome 17 (FTDP-17), which has a microtubule-associated protein tau (MAPT) mutation P301L, tau deposits show different ultrastructures with irregular periodicity from PHFs in AD, while tau deposits in FTDP-17 with MAPT mutation V337M show similar ultrastructures to PHFs in AD [38]. Furthermore, tau protein is modified by multiple post-translational modifications, which might affect the ultrastructural conformation of tau deposits [39]. PHF-tau is abnormally phosphorylated in the brain in AD. Immunoblots of brain homogenates from patients with AD show high molecular weight bands and distinct physical and chemical properties reflecting hyperphosphorylation, although no ultrastructural difference is observed between hyperphosphorylated tau fibrils isolated from AD brains and recombinant unphosphorylated tau fibrils seeded with AD PHFs [40]. In addition to post-translational modification, *cis-trans* conformational isomerization of phosphorylated threonine 231-proline of tau protein plays a role in the accumulation of pathogenic tau in AD [41].

### 2.4. Methods for Histopathological Staining of Tau Deposits

In the progression of neurofibrillary changes at the neuronal cytoskeleton level, it is proposed that the accumulation of soluble phosphorylated tau in cell bodies causes the formation of intracellular classical neurofibrillary tangles [42]. After neuronal death, the abnormal hyperphosphorylated tau finally appears as extracellular ghost tangle, which is seen as loosely packed pale eosinophilic fibrils on hematoxylin and eosin (H&E) staining.

Gallyas silver staining is a classical method for visualizing tau related pathology, including neurofibrillary tangles, neuropil threads, dystrophic neurites, tufted astrocytes, astrocytic plaques, coiled body, and argyrophilic threads. However, this method is insensitive to Pick’s bodies [43,44,45]. Another gold standard method for staining tau related pathology is tau immunohistochemistry using AT8 anti-tau antibody, which recognizes phosphorylated tau at Ser202 and Thr205 [46]. Both methods are now commonly used for the classification of Braak stages at postmortem examination [2,47,48]. Although both methods can detect abnormal neurofibrillary changes at the intraneuronal and neuroanatomical levels, they identify partially different structures. Comparative studies of both methods demonstrated that Gallyas silver staining was sensitive to aggregated fibrillary material like PHFs, while AT8 immunohistochemistry can detect not only aggregated hyperphosphorylated tau but also non-argyrophilic pretangles that contain a mixture of granular tau and small bundles of straight filaments tightly arranged in parallel as observed by electron microscopy [49]. However, AT8 immunohistochemistry is insensitive to late ghost tangles [42,47]. Intraneuronal neurofibrillary tangles are identifiable with equal clarity by both methods. Braak stages (I to VI) were originally determined by Gallyas silver staining, and thereafter revised based on AT8 tau immunohistochemistry [2,47]. Recently, pretangle stages (Stage a, b, c, 1a, and 1b) were added to the original Braak stages [50,51]. According to this staging system, AT8-positive soluble tau begins in the brainstem before the age of 30 years, most frequently in the locus coeruleus, and then spreads to the transentorhinal cortex and develops into intraneuronal Gallyas-positive neurofibrillary tangles [51,52]. Comparative analyses of several different staining methods indicated that β-sheet binding compounds can recognize highly aggregated fibrillary deposits, which are positive with Gallyas silver staining, but not sensitive to AT8-positive pretangle [53,54,55,56]. Autoradiography is a reliable technique to estimate the binding ability and specificity of radiotracers for a wide spectrum of targets at tracer concentrations [10,11]. Binding characteristics of tau radiotracers have been validated by using this method.

## 3. *In Vitro* Binding Characteristics of Tau Radiotracers

Reported tau radiotracers share β-sheet binding properties. The binding of tracers to tau aggregates disappeared after formic acid pretreatment, which disrupts β-pleated sheet structure [55,57,58,59]. Tau radiotracers are designed to show specificity for tau aggregates, because various protein misfolding lesions share common cross β-sheet structure [31]. The β-sheet binding ligands tend to bind to the white matter, because the myelin basic protein in the white matter predominantly forms β-sheet structures [60,61]. Since tau deposits in non-AD conditions also appear in the subcortical regions including the white matter, basal ganglia, and brainstem, tau radiotracers ideally require low off-target binding in these regions. Different chemotype classes of tau radiotracers might show different characteristics of binding to heterogeneous tau deposits in the human brain. PET radiotracers are usually labeled with carbon-11 or fluorine-18. Carbon-11 has a short half-life (t_1/2_ = 20.4 min), which limits its use to centers with an on-site cyclotron and to clinical applications. Fluorine-18 (t_1/2_ = 109.8 min) is suitable for centralized production and regional distribution in the brain for routine clinical use as demonstrated by ^18^F-labeled amyloid PET radiotracers [62].

### 3.1. ^18^F-FDDNP

A naphthylethylidene derivative, ^18^F-FDDNP, was the first PET radiotracer to be applied in clinical PET imaging of tau pathology in patients with AD. Since FDDNP is a highly fluorescent compound, FDDNP clearly stains neurofibrillary tangles. This compound also stains senile plaques, prion plaques, and cerebral amyloid angiopathy, but not globose tangles in PSP and Pick’s bodies [63,64]. In *in vitro* autoradiography of AD brain sections, ^18^F-FDDNP binds diffusely in the neocortex and hippocampus [65,66], indicating that ^18^F-FDDNP binds to both amyloid plaques and neurofibrillary tangles. However, several other reports suggest insufficient binding affinity of FDDNP for *in vivo* detection of neurofibrillary tangles and senile plaques [67,68]. Interestingly, ^18^F-FDDNP binding to the neocortex of AD brains is not displaced by classical amyloid staining dyes such as Congo Red and Thioflavin-T [66]. Furthermore, FDDNP binding does not compete with ^3^H-PiB in AD brain homogenates, indicating the existence of multiple binding sites in AD brains [69].

### 3.2. ^11^C-PBB3

A pyridinyl-butadienyl-benzothiazole derivative, ^11^C-PBB3, has been developed as a unique chemotype class tau PET radiotracer. This tracer was developed for imaging a broad spectrum of tau deposits [70]. PBB3 stains tau deposits including neurofibrillary tangles, Pick’s bodies, tufted astrocytes, astrocytic plaques, coiled bodies, and argyrophilic threads, but also amyloid plaques. *In vitro* autoradiography also demonstrates ^11^C-PBB3 binding to neurofibrillary tangles in AD brain sections.

### 3.3. ^18^F-T808 and ^18^F-AV1451 (as Known as T807)

A benzo[4,5]imidazo[1,2-a]pyrimidine derivative, ^18^F-T808, and a pyridoindole derivative, ^18^F-AV1451 (also known as T807), have been reported as selective tau PET radiotracers [71,72]. As observed in other tau PET tracers, these tracers also show selective labeling of tau pathology with a laminar distribution in the neocortex of AD brain sections [71,72]. Further validation studies of ^18^F-AV1451 autoradiography using postmortem tissues show a strong binding of ^18^F-AV1451 in AD brain sections containing PHF-tau, but no remarkable binding to non-AD tau deposits including those in Pick’s disease, CBD, PSP, or to amyloid-β, α-synuclein, and TDP-43 lesions [73]. However, the binding ability of ^18^F-AV1451 to non-AD lesions is still under debate.

### 3.4. THK Arylquinoline Series

#### 3.4.1. ^18^F-THK523

Arylquinoline derivatives, BF158 and BF170, were initially identified as leading compounds of tau PET probe nearly a decade ago [58,74,75]. These fluorescent compounds clearly stain neurofibrillary tangles, neuropil threads, and dystrophic neurites in AD brain sections. ^18^F-THK523 (^18^F-BF242) was developed as the first ^18^F-labeled arylquinoline derivative [76]. *In vitro* autoradiography of AD brain sections demonstrates a laminar distribution of ^18^F-THK523 in the neocortex, which is consistent with Gallyas silver staining, but not with amyloid-β immunostaining or ^11^C-PiB autoradiography [68,76]. Interestingly, ^18^F-THK523 autoradiography shows a striking similarity to Gallyas silver-positive, but AT8-negative clusters of neurofibrillary tangles in the pre-α of the entorhinal cortex, reflecting late ghost tangles [68]. These observations support that ^18^F-THK523 recognizes argyrophilic fibrillary tau filaments. On the other hand, THK523 failed to stain non-AD tau lesions such as Pick’s bodies and globose tangles in Pick’s disease, CBD, and PSP [77].

#### 3.4.2. ^18^F-THK5105, ^18^F-THK5117, and ^18^F-THK5351

**Figure 3:**
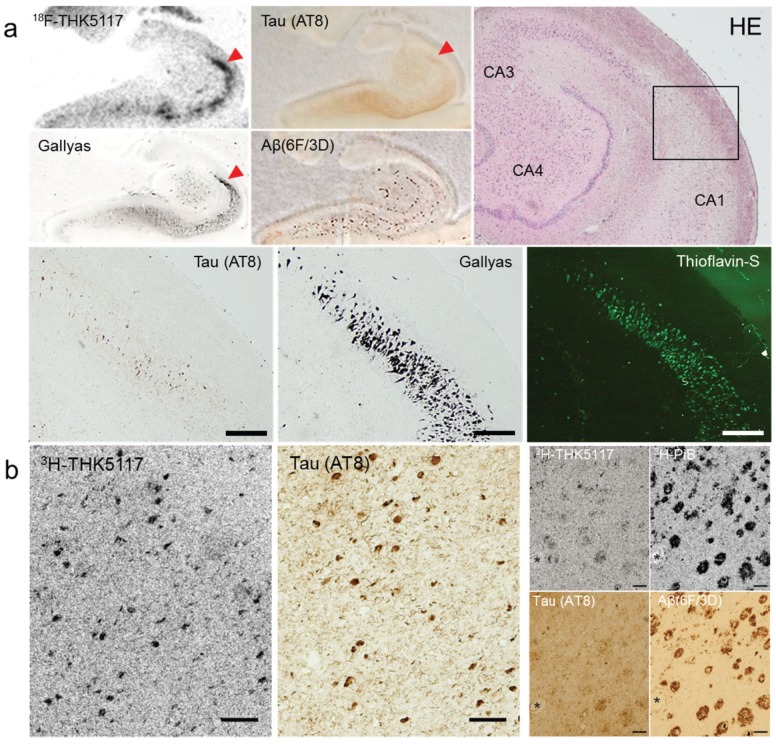
(**a**) *In vitro* autoradiography of ^18^F-THK5117 and tau/amyloid immunohistochemistry, H&E staining, Gallyas silver staining, and thioflavin-S fluorescence staining in the hippocampus of AD brain sections. Scale bars: 400 μm. (**b**) Microscopic observation of ^3^H-THK5117 and ^3^H-PiB labeled sections after photo emulsion treatment and tau/amyloid immunohistochemistry in adjacent sections. (asterisks indicate same blood vessel, scale bars 100 μm).

Compound optimization of arylquinoline derivatives resulted in the development of three ^18^F-labeled radiotracers, ^18^F-THK5105, ^18^F-THK5117, and ^18^F-THK5351. These compounds possess higher binding affinities for tau aggregates in AD brains than ^18^F-THK523, and preferable pharmacokinetics without defluorination *in vivo* [78]. As observed in ^18^F-THK523, these new tracers also demonstrate high selectivity for tau pathology in AD brains [79]. As shown in Figure 3a, ^18^F-THK5117 preferentially accumulates in the CA1 of the hippocampus containing high density of late ghost tangles in AD brain sections. Microautoradiography using ^3^H-labeled compounds demonstrates selective binding ability of THK5117 to neurofibrillary pathology (Figure 3b). The binding of THK5117 to tau aggregates is dependent on the β-sheet structure of PHF-tau, but independent of tau isoform composition and phosphorylation state [59], suggesting that the phosphorylation state may not affect the ultrastructure of tau aggregates and the binding sites of ligands. THK5117 labels both intracellular and extracellular tangles, but not pretangles, as previously observed in a β-sheet binding compound, thiazine red [80]. Therefore, THK5117 seems to be insensitive to pretangle tau deposits that are observed in normal brain before the age of 30 years. ^18^F-THK5105 and ^18^F-THK5117 shows substantial white matter binding, which may lead to misinterpretation of the PET images. For this reason, ^18^F-THK5351 was additionally developed to reduce the white matter binding of arylquinoline derivatives [81]. Compared with ^18^F-THK5117, ^18^F-THK5351 showed higher signal-to-background ratio in *in vitro* autoradiography using human brain sections.

**Figure 4:**
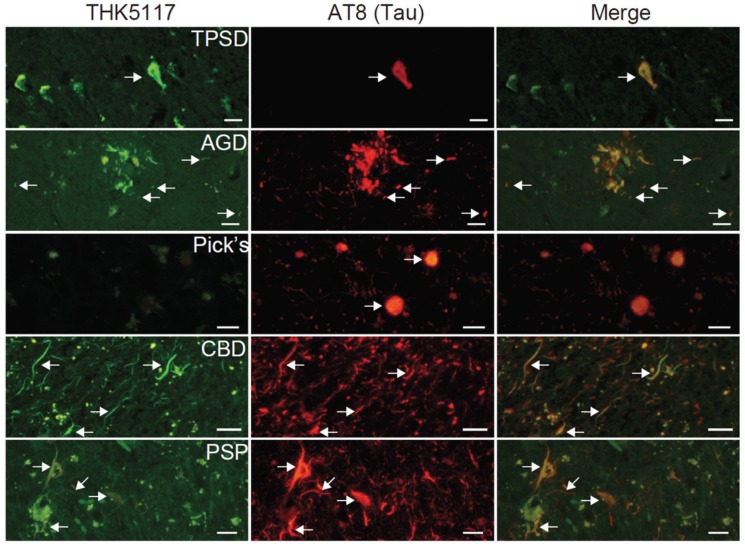
Fluorescence staining of THK5117 in various tauopathies such as tangle predominant senile dementia (TPSD), argyrophilic grain disease (AGD), Pick’s disease, corticobasal degeneration (CBD), and progressive supranuclear palsy (PSP). Scale bar: 20 μm. Arrows indicate the same tau deposits for each brain sample.

THK5117 stains neurofibrillary tangles in TPSD, argyrophilic grains in AGD, argyrophilic threads in CBD, neurofibrillary tangles and globose tangles in PSP, but not Pick’s bodies in Pick’s disease (Figure 4). THK5117 failed to label α-synuclein and TDP-43 containing lesions.

### 3.5. Other Classes

After the discovery of benzimidazole derivatives as potential candidates for tau radiopharmaceuticals [58,75], several groups have been investigating the binding characteristics of related compounds to tau pathology. Lansoprazole derivatives have been reported to label tau regions in AD and PSP brain sections [82,83], although the details of the binding characteristics of these derivatives remain unknown. ^125^I-labeled benzimidazole derivatives without dimethyl amino group have also been reported as potential selective tau PET tracer candidates [84]. The pattern of binding was very similar to AT8 tau immunohistochemistry. These radiotracers have not been tested in humans.

## 4. Current Status of Tau PET Imaging in Clinical Studies

Several tau PET tracers have been tested in humans. However, none of these radiotracers has been fully validated yet.

### 4.1. ^18^F-FDDNP

^18^F-FDDNP PET study demonstrated high tracer uptake in the medial temporal and neocortex of patients with AD [85], supporting that ^18^F-FDDNP binds to both amyloid plaques and neurofibrillary tangles. Correlation analysis of imaging with autopsy finding shows close association between *in vivo* cortical binding of FDDNP and the density of amyloid and tau deposits [86,87]. ^18^F-FDDNP retention is also observed in various type of diseases including prion disease, frontotemporal dementia, Down’s syndrome, PSP, and in American football players with suspected CTE [88,89,90,91,92,93,94]. However, there is some conflict between PET findings and *in vitro* binding data showing negligible binding to Pick’s bodies and globose tangles in frontotemporal dementia and PSP [63,64]. A radiolabeled metabolite of ^18^F-FDDNP is reported to cross the blood–brain barrier [95].

### 4.2. ^11^C-PBB3

^11^C-PBB3 PET study demonstrated significant tracer retention in the hippocampus of patients with AD, suggesting that ^11^C-PBB3 selectively binds to tau deposits in AD. In addition, a case with corticobasal syndrome (CBS) showed elevated ^11^C-PBB3 retention in the basal ganglia, and the spatial pattern of PBB3 retention was consistent with that of brain atrophy [70]. Ongoing imaging-autopsy studies will validate these initial findings. A radiolabeled metabolite of ^11^C-PBB3 can cross the blood–brain barrier; therefore, some special technique is required for the quantitative analysis of PET data [96,97,98].

### 4.3. ^18^F-AV1451 (^18^F-T807)

Initial human PET studies successfully demonstrated high retention of ^18^F-AV1451 in regions known to contain high density of tau deposits in patients with AD with low white matter retention. A strong association was observed between the amount of radiotracer and the severity of dementia [99,100]. A large multicenter study of ^18^F-AV1451 is ongoing. A case report on posterior cortical atrophy (visual variant of AD) demonstrated that ^18^F-AV1451 binding in the posterior brain regions correspond to reduced ^18^F-FDG uptake and clinical symptom [101]. Case reports of non-AD tauopathies such as frontotemporal dementia with MAPT mutation P301L, suspected CTE, PSP, and CBS also demonstrated elevated binding of ^18^F-T807 in frequent areas of tau aggregates in those conditions [102,103,104]. However, these *in vivo* findings have not yet been fully validated by imaging-autopsy studies [73].

### 4.4. ^18^F-THK Arylquinoline Series

**Figure 5:**
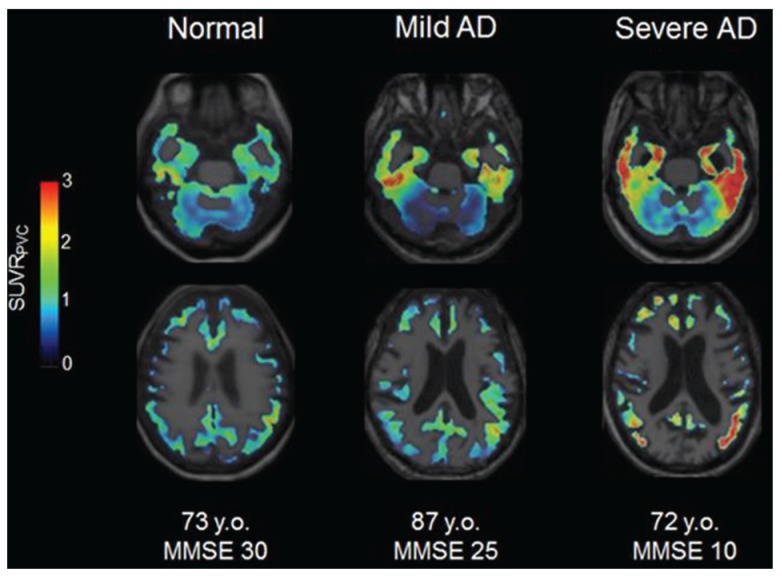
^18^F-THK5117 PET imaging in a normal elderly subject (Mini Mental State Examination (MMSE) 30, 73 y.o.), patient with mild AD (MMSE 25, 87 y.o.) and patient with severe AD (MMSE 10, 72 y.o.).

Although ^18^F-THK523 imaging failed in visualizing the tau deposits clearly in patients with AD [105], ^18^F-THK5105 and ^18^F-THK5117 demonstrated a robust difference between patients with AD and healthy control subjects in brain regions known to contain high density of tau deposits (Figure 5). These tracer bindings were correlated with brain atrophy and severity of dementia [106]. ^18^F-THK5117 showed better pharmacokinetics and higher signal-to-background ratio than ^18^F-THK5105 [59]. The regional distributions of these radiotracers were substantially different from that of ^11^C-PiB in the same population. ^18^F-THK5117 positive voxels were closer to the white matter than ^11^C-PiB positive voxels, which may reflect the binding of ^18^F-THK5117 to tau deposits in layer V of the neocortex. Recent longitudinal studies of ^18^F-THK5117 demonstrated that the annual changes in tracer binding were significantly increased in the temporal cortex of patients with AD, which was closely associated with the annual changes in cognitive decline [107]. Recent ^18^F-THK5351 PET studies demonstrated greater specific signals in the frequent sites of tau deposition and lower non-specific binding in the white matter than ^18^F-THK5117 [81].

## 5. Conclusions

This paper described *in vitro* binding characteristics of reported tau PET radiotracers, and status of clinical PET imaging (Table 1). Tau PET imaging will provide the longitudinal information about tau accumulation in the human brain during aging and in neurodegenerative conditions. The combination of tau and amyloid PET imaging will enable accurate diagnosis of AD, early detection of individuals at-risk who have AD neuropathology, and help in understanding when, where, and how tau tangles interact with amyloid plaques in the brain. Recent clinical studies suggest that the neocortical tau accumulation yields clinical symptom of dementia [108,109,110]. Therefore, preventing the spread of tau pathology to the neocortex would be a promising strategy for the prevention of dementia [111]. Assessing the regional distribution of tau deposits would play a critical role in evaluating therapeutic efficacy of anti-dementia drugs.

**Table 1:** *In vitro* binding and *in vivo* characteristics of reported tau PET radiotracers.

		FDDNP	PBB3	AV-1451 (T-807)	THK-5117	THK-5351
Developer/License		UCLA	NIRS	Avid	Tohoku University/GEHC	
Radionuclide		^18^F	^11^C	^18^F	^18^F	^18^F
Molecular weight		293.4	308.4	262.1	326.4	327.4
*In vitro* binding	*K*_d_ (nM) (AD brain) *^a^	N.D. *^b^	2.6 *^c^	14.6 *^c^	11.5 *^d^	2.9 *^d^
	Selectivity Tau/Aβ (autoradiography)	N.D. *^b^	40–50	> 25	30	> 30
*In vivo*	Maximum brain uptake in mice	6.23	1.92	4.43	6.06	4.35
	Maximum/30 min uptake ratio in mice	3.08	17.5	7.15	10.4	20.7
	Brain uptake of radioactive metabolites	Yes	Yes	No	No	No
	WM binding	High	Low	Low	High	Low
References		[85]	[70,96]	[72,99]	[59,78]	[81]

*^a^ All ligands reported only *K*_d_ values for AD brains, not for non-AD tauopathies brains. *^b^ N.D. = not determined. *^c^ *K*_d_ values were determined by *in vitro* autoradiography using AD brain sections. *^d^ *K*_d_ values were determined by *in vitro* saturation binding assay using AD brain homogenates.
